# Hybrid deep learning model for brain age prediction using time-distributed convolutional and bidirectional LSTM networks

**DOI:** 10.1038/s41598-026-54198-5

**Published:** 2026-06-12

**Authors:** Eslam Mahmoud, Nada M. Elshennawy, Amr Elkholy

**Affiliations:** Department of Computer and Control Engineering, Faculty of Engineering, Tanta University, Tanta, Egypt

**Keywords:** Brain age prediction, Neuroimaging, OpenBHB, Deep learning, Magnetic Resonance Imaging (MRI), Computational biology and bioinformatics, Mathematics and computing, Neurology, Neuroscience

## Abstract

Brain age prediction has gained significant attention due to its strong correlation with neurological and cognitive disorders. The discrepancy between an individual’s chronological age and their predicted brain age–known as the Brain Age Gap–has been linked to conditions such as schizophrenia, Alzheimer’s disease, cognitive decline, and lifestyle factors like stress and poor health. A positive Brain Age Gap is often associated with accelerated aging and neurodegeneration, highlighting the need for precise and reliable estimation methods. In this study, we propose a novel deep learning model that incorporates time-distributed, convolutional and bidirectional LSTM layers for brain age estimation. Using MRI data from the OpenBHB dataset, processed through Voxel-Based Morphometry (VBM), our model undergoes rigorous preprocessing, including outlier detection, data augmentation, and MRI slice selection, to enhance learning efficiency. The model is optimized with the Adam optimizer with a scheduled learning-rate decay and evaluated using Mean Absolute Error (MAE) and $R^{2}$ Score. Experimental results demonstrate that our model achieves an MAE of 3.1573 years, outperforming previous methods and improving brain age prediction accuracy. These findings underscore the importance of advances in deep learning and data preprocessing in enhancing brain age estimation.

## Introduction

Brain age prediction is the process of estimating an individual’s brain age using neuroimaging modalities such as Magnetic Resonance Imaging (MRI), Computed Tomography (CT), Functional MRI (fMRI), Positron Emission Tomography (PET), Electroencephalography (EEG), and Magnetoencephalography (MEG). A key outcome of this process is the Brain Age Gap (BAG), defined as the difference between an individual’s predicted brain age and their chronological age. This gap acts as a critical neuroimaging biomarker, reflecting deviations from normative brain aging trajectories. A positive BAG (i.e., the brain appears older than the individual’s actual age) has been associated with accelerated neurodegeneration, whereas a negative BAG may suggest neuroprotection or resilience to aging. Numerous studies have demonstrated associations between abnormal BAG values and a wide range of neurological and psychiatric conditions, including Alzheimer’s disease^1,2^, Parkinson’s disease^3,4^, Multiple Sclerosis^5^, Schizophrenia^6^, Major Depressive Disorder^7^, Traumatic Brain Injury^8^, and HIV-Associated Neurocognitive Disorder^9^.

Previous studies have reported elevated Brain Age Gap (BAG) values in several neurological and psychiatric disorders compared with cognitively normal individuals. In a large cross-sectional analysis^10^, it was reported that cognitively normal participants exhibited BAG values close to zero (0.122 ± 3.039 years). In contrast, neurodegenerative conditions showed substantially higher BAG values, including Alzheimer’s disease (3.242 ± 6.635 years) and mild cognitive impairment (2.06 ± 5.62 years)^10^. Similarly, several psychiatric disorders were associated with elevated BAG, including schizophrenia (2.068 ± 3.341 years) and bipolar disorder (1.913 ± 4.051 years)^10^.

Prior studies have also shown that a higher BAG is significantly associated with elevated levels of neurodegeneration-related biomarkers–such as amyloid-$\beta$, phosphorylated tau (pTau), and neurofilament light chain (NfL)–detected in cerebrospinal fluid, plasma, and through PET imaging^11^. Moreover, BAG correlates with cardiovascular and lifestyle-related risk factors–including obesity, waist-to-hip ratio, smoking, hypertension, and alcohol consumption–even among cognitively healthy, middle-aged individuals^1^. Collectively, these findings highlight the utility of BAG as a non-invasive, quantitative biomarker for the early detection, risk stratification, and longitudinal monitoring of both neurodegenerative and psychiatric diseases, supporting its potential role in early diagnosis, disease progression tracking, and intervention planning.

Nevertheless, accurate brain age estimation remains challenging due to biological variability, imaging heterogeneity, and the need for large, demographically diverse datasets. Addressing these complexities is crucial for improving the reliability and clinical utility of brain age prediction models.

Brain age prediction relies on diverse neuroimaging datasets, each offering unique insights into brain structure and aging patterns:

- UK Biobank^12,13^: The largest dataset, containing neuroimaging and biomedical data from 500,000 participants in the UK, aged 40 to 69 years, collected between 2006 and 2010.
- IXI^14^: A dataset of 600 MRI scans from healthy individuals aged 20 to 80 years, acquired from three London hospitals using different MRI scanners.
- ADNI^15–17^: A longitudinal dataset with 2,000 participants aged 50 to 90 years, including individuals with Alzheimer’s disease and mild cognitive impairment. Data collection began in 2004 and continues, with the latest phase expected to conclude in 2027^17^.
- OASIS^18–20^: Comprising 1,099 participants across three phases (OASIS-1^19^, OASIS-2, and OASIS-3^20^), this dataset focuses on structural brain imaging for aging and neurodegenerative research.
- GSP^21^: Collected by Harvard University and Massachusetts General Hospital, this dataset includes 1,570 participants aged 18 to 35 years, emphasizing young adult brain structure and function.
- OpenBHB^22^: A large-scale dataset with over 5,000 records from 10 public datasets (including IXI, ABIDE, CoRR, GSP, and others), covering healthy individuals aged 6 to 88 years. It includes demographic information, brain volume metrics–such as White Matter Volume (WMV), Gray Matter Volume (GMV), Cerebrospinal Fluid Volume (CSFV), and Total Intracranial Volume (TIV)–as well as preprocessed MRI images in Voxel-Based Morphometry (VBM), Surface-Based Morphometry (SBM), and quasi-raw formats.

These datasets serve as the foundation for brain age prediction models that leverage deep learning to estimate brain age and assess cognitive health. Key architectures used in these models include Simple Fully Convolutional Networks (SFCN)^23^, Convolutional Neural Networks (CNNs)^24–27^, Transformers^27^, and Autoencoders^28,29^. Building on these datasets and neural architectures, researchers aim to enhance the accuracy of brain age prediction. This progress facilitates early diagnosis, monitoring of neurodegenerative diseases, and the development of personalized medicine.

This paper presents a novel deep learning model with optimized preprocessing techniques, applied to the OpenBHB^22^ dataset processed using Voxel-Based Morphometry (VBM). Our model outperforms existing approaches on the same dataset by achieving a lower Mean Absolute Error (MAE). The paper begins with a discussion of related work in Section 2, reviewing existing brain age prediction models and their applications across various datasets. It then introduces the proposed model, detailing its architecture and key enhancements in Section 3. The Experiments section focuses on the implementation process and dataset preparation, as described in Section 4. The results of our model, along with a comparative analysis against existing approaches, are discussed in Section 5. Finally, Section 6 concludes the study by summarizing the key findings and outlining potential directions for future research in brain age prediction.

## Related work

The SFCN^23^ (Simple Fully Convolutional Network) model is a deep learning architecture designed for brain age prediction. It achieved a Mean Absolute Error (MAE) of 2.14 years on the UK Biobank^12,13^ dataset, which included 14,503 records from individuals aged 42 to 82 years. The model also achieved an MAE of 2.90 years on the Predictive Analytics Challenge (PAC) 2019 dataset, which contained 2,638 records from participants aged 14 to 94 years. The model employs a fully convolutional design, comprising multiple layers for feature extraction and prediction. It includes five repeated blocks containing 3D convolution, batch normalization, max pooling, ReLU activation, average pooling, dropout, and a softmax output layer.

The CNN model proposed in^24^ for brain age prediction achieved a Mean Absolute Error (MAE) of 2.7 years on the UK Biobank^12,13^ dataset, using 16,998 records. The model processes brain images through an initial convolutional layer, followed by six bottleneck layers, an average pooling layer, and a fully connected layer, which generates the final predicted brain age.

Another study^25^ proposed a dual-stream CNN model integrating T1-weighted and time-of-flight magnetic resonance angiography (TOF MRA) images to predict brain age. The model was evaluated on 2,074 participants from the Study of Health in Pomerania (SHIP) dataset, which includes both image types. The dataset was split into 1,340 records (65%) for training, 334 (15%) for validation, and 400 (20%) for testing. The architecture uses two parallel CNNs, one for each modality, each with four convolutional layers featuring batch normalization, max pooling, ReLU activation, average pooling, dropout, and a dense layer for age estimation. Outputs from both CNNs are merged using a linear regression model to improve accuracy. The model achieved a Mean Absolute Error (MAE) of 3.85 years on the test set, highlighting the potential of multimodal imaging in brain age estimation.

Another research^27^ integrates Convolutional Neural Networks (CNNs) and Transformers to analyze relationships between paired MRI images. It consists of a feature-extraction stage in which two CNN backbones process the input images x and extract meaningful representations. These features are then divided into patches and passed as tokens to a Transformer-based relation regression module. The Transformer computes various relationships between the extracted features, including sums, differences, maximums, and minimums. This hybrid CNN-Transformer architecture enables efficient learning from large-scale brain imaging data. The study used 6,049 MRI records from 8 datasets spanning ages 0 to 97 years and achieved a Mean Absolute Error (MAE) of 2.38 years, demonstrating the effectiveness of combining CNNs with Transformers for brain age prediction.

In^28^, a model based on an Autoencoder for brain age prediction is proposed. This model employs an encoder-decoder architecture with a latent-space disentanglement module that separates age-related features for age estimation while preserving age-unrelated components. The model was trained using the Study of Health in Pomerania (SHIP) dataset and tested on the IXI dataset. The model achieved Mean Absolute Error (MAE) values of 4.95 to 5.77 years during training and 6.97 to 8.65 years during testing, highlighting the challenges of generalizing brain age estimation across different datasets.

Several studies have explored brain-age prediction using the OpenBHB dataset^22^. One study^30^ achieved an MAE of 4.55 years on its internal test set. Another study^29^ developed a model based on an Autoencoder and a fully connected neural network with ReLU activation and batch normalization, achieving an MAE of 4.554 years on the training dataset. Additionally, another study^31^ implemented a regression-based approach. This study utilized the training and validation sets containing 3965 records (after excluding some from the original 3984 records), with 3172 records (80%) for training and 793 records (20%) for testing. The model achieved an MAE of 3.25 years on the test dataset, demonstrating improved accuracy compared to other models.

## Proposed model

**Fig. 1:**
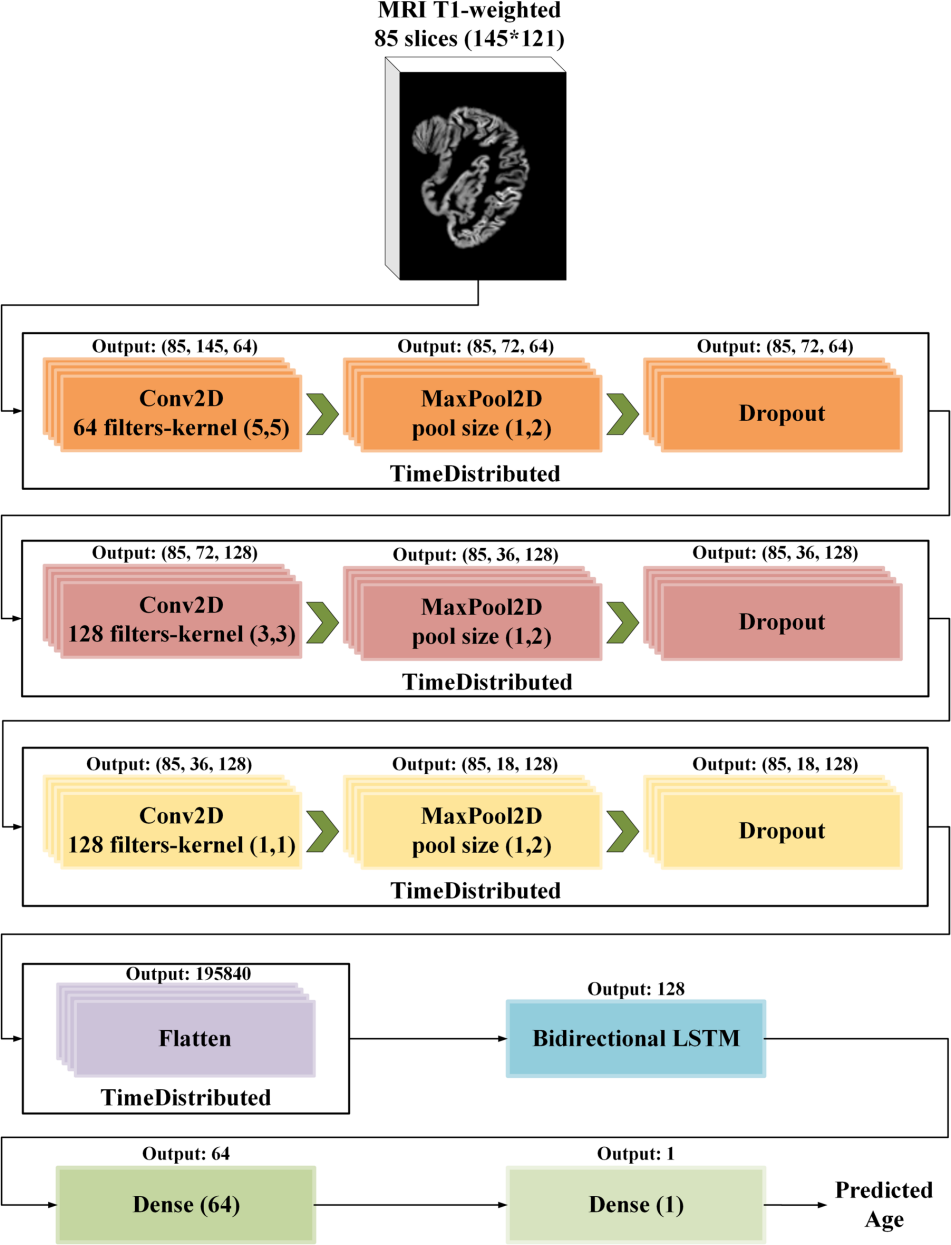
Proposed brain age prediction model.

The proposed model, illustrated in Fig. 1, uses T1-weighted MRI images and leverages a combination of Convolutional Neural Networks (CNNs) and Long Short-Term Memory (LSTM) networks to effectively extract both spatial and temporal features from MRI slices, thereby ensuring a robust and accurate age-prediction system. The input to this model consists of MRI scan slices, which are processed sequentially to capture anatomical variations associated with aging. Figure 1 also illustrates the data flow within the model, providing a clear visual representation of how input MRI slices are processed through the feature-extraction and prediction stages, thereby facilitating a better understanding of the model’s internal structure. Table 1 presents the detailed architecture of the proposed model, illustrating the transformation of data dimensions at each stage. It highlights how spatial and temporal features are progressively extracted through TimeDistributed convolutional and pooling layers, followed by sequential modeling via a Bidirectional LSTM. Finally, dense layers integrate the learned representations to generate the predicted brain age. This architecture is carefully structured to leverage the powerful feature-extraction capabilities of CNNs while leveraging the sequential processing strengths of LSTMs, making it highly effective for modeling brain structures and their age-related changes.

The proposed architecture employs a 2D slice-based design enhanced with TimeDistributed layers to model inter-slice dependencies within 3D MRI volumes. While conventional 3D architectures directly process volumetric data, they are computationally expensive and memory-intensive. In contrast, the TimeDistributed framework allows the model to extract spatial features from individual slices while maintaining sequential relationships across them, effectively capturing partial 3D context. This design offers several advantages, including reduced computational cost, improved training efficiency, and compatibility with high-resolution input data under limited hardware resources. Consequently, the TimeDistributed configuration provides a practical and scalable compromise between model complexity and performance, enabling robust brain age prediction across heterogeneous, multi-site MRI data.

**Table 1:** Model architecture and corresponding output shapes at each stage.

Component	Output Shape	Description
**Input** (T1-weighted MRI)	(85, 145, 121)	85 slices, each of size 145$\times$121.
**Block 1**: TimeDistributed of 2D convolutional with 64 filters and kernel size (5$\times$5))	(85, 145, 64)	Each 2D convolutional kernel scans the spatial dimensions (85$\times$145) while treating all 121 slices as input channels. This operation produces 64 spatial feature maps per slice with padding=’same’.
**Block 2**: TimeDistributed of MaxPool2D (1$\times$2)	(85, 72, 64)	Pooling halves the width (145 $\rightarrow$ 72) while preserving height and channel count.
**Block 3**: TimeDistributed of Dropout	(85, 72, 64)	Applies regularization without changing spatial dimensions.
**Block 4**: TimeDistributed of 2D convolutional with 128 filters and kernel size (3$\times$3)	(85, 72, 128)	Each 2D convolutional kernel scans the spatial dimensions (85$\times$72) of every slice while treating the previous feature maps as input channels. This operation generates 128 spatial feature maps per slice with padding=’same’.
**Block 5**: TimeDistributed of MaxPool2D (1$\times$2)	(85, 36, 128)	Pooling halves the width (72 $\rightarrow$ 36) while preserving height and channel count.
**Block 6**: TimeDistributed of Dropout	(85, 36, 128)	Applies regularization without changing spatial dimensions.
**Block 7**: TimeDistributed of 2D convolutional with 128 filters and kernel size (1$\times$1)	(85, 36, 128)	Each 2D convolutional kernel scans the spatial dimensions (85$\times$36) of every slice while treating the previous feature maps as input channels. This operation generates 128 spatial feature maps per slice with padding=’same’.
**Block 8**: TimeDistributed of MaxPool2D (1$\times$2)	(85, 18, 128)	Pooling halves the width (36 $\rightarrow$ 18) while preserving height and channel count.
**Block 9**: TimeDistributed of Dropout	(85, 18, 128)	Applies regularization without changing spatial dimensions.
**Block 10**: TimeDistributed of Flatten	195840	Each slice is flattened into a 1D feature vector (85$\times$18$\times$128 = 195840).
**Block 11**: Bidirectional LSTM with 64 units	128	Outputs 64 features per direction (64$\times$2 = 128).
**Block 12**: Dense with 64 neurons	64	Fully connected layer producing a compact representation.
**Block 13**: Dense with single neuron	1	Final regression output – predicted brain age.

The first component of the model comprises three convolutional layers, each wrapped in a TimeDistributed layer to ensure consistent application of CNN operations across multiple MRI slices. These convolutional layers are essential for learning spatial features from the input and for capturing structural patterns, such as brain tissue morphology, cortical thickness, and other characteristics associated with aging.

The first convolutional layer employs 64 filters with a kernel size of (5, 5), followed by a MaxPooling layer with a pool size of (1, 2) and a dropout layer to reduce overfitting and enhance generalization. The second convolutional layer increases the filter count to 128 and uses a smaller kernel size of (3, 3), enabling the model to extract more refined features while maintaining the same pooling and dropout configurations. The third convolutional layer retains 128 filters but reduces the kernel size to (1,1), focusing on fine-grained spatial details to capture subtle structural variations relevant to age prediction.

Each convolutional layer is followed by MaxPooling, which reduces the spatial dimensions of the feature maps, and dropout, which improves robustness by randomly deactivating neurons during training.

After feature extraction, the model flattens the feature maps using another TimeDistributed layer, converting them into a structured sequence of feature vectors while preserving the sequential nature of the MRI slices. These feature vectors are then processed by a Bidirectional LSTM (Long Short-Term Memory) network, which plays a crucial role in modeling temporal dependencies across MRI slices. Since brain aging is a gradual, progressive process, the LSTM component helps capture long-range dependencies, ensuring that the relationship between slices is fully utilized. The Bidirectional LSTM processes the sequence in both forward and backward directions, allowing it to learn both past and future correlations in the data, leading to a more refined age prediction. This bidirectional approach is particularly useful in medical imaging applications where subtle structural changes need to be analyzed holistically. Following the LSTM layer, the extracted high-level features are passed through two fully connected (Dense) layers, which further refine and process the learned representations. The first Dense layer consists of 64 neurons, serving as a compression layer to distill the most relevant information from the LSTM outputs while reducing redundancy. This layer applies a nonlinear transformation, improving the model’s generalization to unseen MRI scans. Finally, the second Dense layer, consisting of a single neuron, produces the final brain age prediction. This final layer ensures that all the extracted spatial and temporal features contribute to a single, precise numerical estimate of the subject’s brain age.

The following points provide the rationale behind the architectural design of the proposed model:

- We employed a sequence of convolutional layers with kernel sizes of (5,5), (3,3), and (1,1). The (5,5) kernel at the initial stage enables the model to capture broader anatomical structures, such as large cortical patterns and inter-regional dependencies. The (3,3) kernel in the intermediate layer focuses on refining mid-level features, while the (1,1) kernel serves as a channel-wise projection layer, effectively reducing dimensionality and enhancing nonlinearity without affecting spatial resolution^32^.
- The number of convolutional filters in our model was progressively increased from 64 in the first layer to 128 in the second and third layers in order to expand the network’s representational capacity in a controlled manner. Starting with 64 filters is consistent with established CNN architectures such as VGG-16^33^, which begins with 64 filters and systematically doubles them in deeper layers. This design balances early-stage feature extraction with computational efficiency, while the deeper layers with more filters enable the model to learn increasingly abstract and complex representations.
- Although the use of a (1,2) MaxPooling layer is not commonly reported in prior brain MRI models, this choice is motivated by the observation that 2D MRI slices often exhibit anisotropic resolution, with structural variability and detail more pronounced along the vertical axis, particularly in sagittal and coronal planes. For example, in a study analyzing MR texture features, sagittal slices showed nearly 60% sensitivity to slice thickness, compared to 20% in axial and 10% in coronal views^34^. This asymmetric pooling approach enables downsampling along the width while preserving vertical anatomical information, thereby reducing the risk of discarding clinically relevant features.
- Number of neurons in the first fully connected (Dense) layers (64 neurons) was determined through ablation testing, where different configurations (e.g., 32 and 128 neurons) were evaluated. The 64-neuron setting consistently achieved the best performance while keeping the model compact and computationally efficient.

Overall, our model represents a sophisticated integration of CNN-based spatial feature extraction and LSTM-based sequential processing, making it well-suited to handle 3D MRI scans and effectively predict brain age. By combining the strengths of convolutional layers for detecting anatomical structures and LSTM layers for capturing temporal dependencies, the model achieves high accuracy in predicting brain age from MRI scans. Additionally, the incorporation of dropout layers, max pooling, and bidirectional processing ensures robustness, reducing overfitting and improving generalization across different datasets. The proposed approach is particularly advantageous in medical imaging applications, where both spatial and sequential dependencies are crucial for analyzing the complex variations in brain morphology associated with aging.

## Experiments

### OpenBHB dataset and Voxel-Based Morphometry (VBM)

In this study, we initially attempted to access several well-known brain imaging datasets, including UK Biobank^12,13^ and PAC 2019^35^, to broaden our evaluation scope. However, our access requests were denied by the relevant data providers, limiting our ability to include them in this work. Although we successfully obtained access to both the IXI^14^ and OASIS^18–20^ datasets, we found that the total number of samples in each (approximately 600–1,000 subjects) was insufficient to effectively train the proposed deep learning model, which requires a larger dataset to avoid overfitting and ensure reliable generalization.

In contrast, the OpenBHB^22^ dataset offers a substantially larger, more diverse sample, comprising thousands of T1-weighted MRI scans collected from 71 sites. Uniquely, it aggregates data from 10 distinct datasets, capturing a wide range of demographic, clinical, and technical variability. This inherent cross-dataset and cross-site heterogeneity makes it particularly suitable for evaluating the generalizability and robustness of brain age prediction models. Consequently, we focused our experimental evaluation on this dataset, as it provides a realistic, large-scale, and heterogeneous neuroimaging environment–without the need for additional dataset integration.

Furthermore, OpenBHB^22^ was selected not only for its scale and diversity but also for providing publicly available, preprocessed data specifically designed for brain age estimation and biomarker discovery. Our experiments were conducted using the dataset preprocessed with Voxel-Based Morphometry (VBM), a key technique for analyzing structural brain properties. VBM is widely used in neuroimaging as it enables a detailed examination of gray matter volume, density, and concentration across different brain regions. By transforming raw MRI scans into a standardized space, VBM facilitates voxel-wise statistical analysis, making it particularly effective for detecting subtle age-related changes in brain structure. This approach is advantageous for brain age estimation as it captures global and regional aging-related alterations. Additionally, VBM-based features reduce computational complexity while preserving critical anatomical information, making them well-suited for deep learning models.

### Outlier detection and removal

To ensure data reliability, outlier detection and removal were performed as a crucial preprocessing step. Outliers in neuroimaging data can arise due to motion artifacts, scanner inconsistencies, or atypical brain structures, all of which may distort model training and lead to unreliable predictions.

The removal of these outliers significantly altered the dataset distribution, leading to a more balanced and biologically plausible representation of brain aging patterns. By eliminating extreme values, we reduced the risk that our model would learn noise rather than meaningful structural variations associated with age. This approach helped stabilize variance, improve data normality, and enhance model generalization, ultimately resulting in more precise and unbiased brain age predictions. Furthermore, outlier removal contributed to a smoother learning process during training, reducing the likelihood of overfitting to anomalous cases and improving the model’s overall predictive performance on unseen data.

To identify and remove these outliers in the OpenBHB^22^ dataset, we examined the distribution of key brain volume metrics–gray matter volume (GMV), white matter volume (WMV), total intracranial volume (TIV), and cerebrospinal fluid volume (CSFV)–across subjects grouped into 10-year age bins (e.g., 10–19 years, 20–29 years, etc.) and generated histograms for each group. Histogram-based distribution analysis was conducted for each age group. Outliers were identified as extreme observations that were clearly separated from the main distribution and inconsistent with expected anatomical trends within their corresponding age group. When 10-year bins did not provide sufficient resolution to distinguish anomalous values, the analysis was repeated with 1-year bins to improve detection sensitivity.

**Fig. 2:**
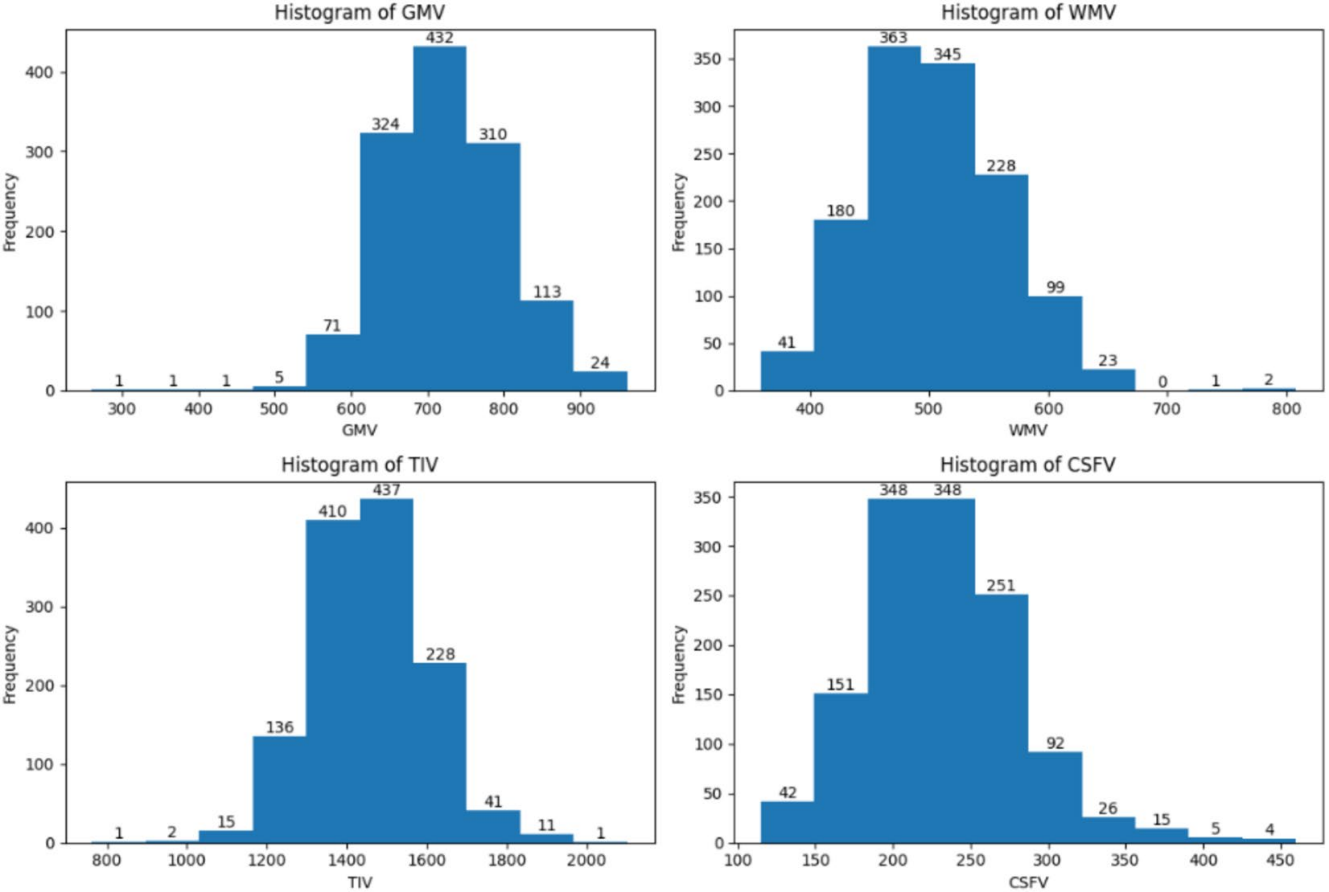
Distribution for ages between 10 and 19.

**Fig. 3:**
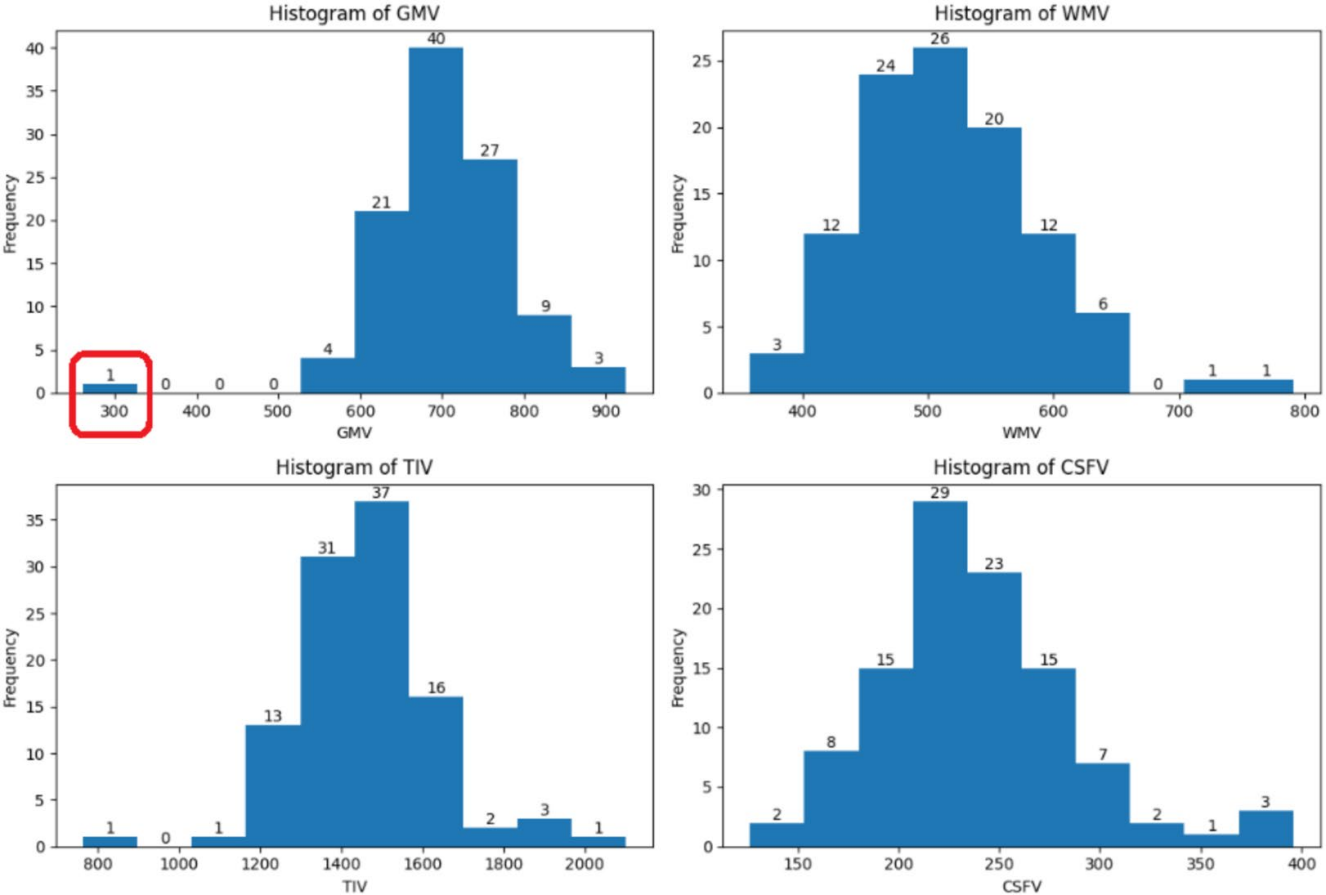
Distribution for ages 18 years and above but below 19 years.

Importantly, removal was conservative and limited to a small number of subjects exhibiting implausible volumetric patterns across one or more metrics. This approach ensured the elimination of corrupted or biologically inconsistent samples while preserving natural inter-subject variability. For example, Figs. 2 and 3 illustrate the distribution of GMV, WMV, TIV, and CSFV for different age groups. Figure 2 represents the distribution for ages between 10 and 19 years, while Fig. 3 provides a more detailed view, focusing specifically on ages 18 years and above but below 19 years. In these distributions, one record is identified as an outlier, highlighting deviations from the dataset’s typical patterns.

Before outlier detection, the OpenBHB^22^ dataset comprised 3,984 records. Using a histogram-based approach across different age groups and brain volume metrics, 33 records were identified as outliers and removed, resulting in a refined dataset of 3,951 records that were used for subsequent training and evaluation. This preprocessing step improved the biological plausibility of the dataset by removing extreme values that could otherwise introduce noise or distort the learning process. By eliminating anomalous samples, the model was better able to capture meaningful structural variations associated with aging, thereby improving stability during training and more reliable brain age predictions.

### Dataset splitting and allocation

Since the publicly available OpenBHB^22^ dataset includes only training and validation datasets, we combined them into a single dataset and then randomly split it into ”Training and Validation” (80%) and ”Test” (20%) sets while maintaining the same age distribution. Furthermore, the ”Training and Validation” set was further divided into ”Training” (80%) and ”Validation” (20%) subsets, ensuring a balanced representation of age groups. Table 2 provides detailed information about the dataset after outlier detection and removal, including its division into training, validation, and test datasets.

**Table 2:** DataSet after outlier removal.

	Total data	Train data	Validation data	Test data
Size	3951	2528	632	791
Age Mean	24.83	24.89	24.73	24.75
Age Std	14.20	14.20	14.29	14.10
Males	2068 (52.34%)	1324 (52.37%)	332 (52.53%)	412 (52.09%)
Females	1883 (47.66%)	1204 (47.63%)	300 (47.47%)	379 (47.91%)

### Data augmentation

In the OpenBHB^22^ dataset, there was a noticeable imbalance in the distribution of records across different age groups. Specifically, the number of records for subjects younger than 10 years and older than 30 years was significantly lower than for those aged 10 to 30. This imbalance could lead to biased model training, where the model becomes overly attuned to the dominant age range and struggles to generalize to underrepresented age groups.

To address this issue, we applied data augmentation techniques specifically to these underrepresented age groups (younger than 10 years and older than 30), but only within the training and validation datasets, ensuring that the test dataset remained untouched for fair evaluation. For each selected record, six additional samples were generated using anatomically preserving spatial transformations applied to the MRI slices: Flip, Mirror, Flip Mirror, Rotate 10 degrees, Rotate 20 degrees, and Rotate 30 degrees. These transformations were intentionally restricted to moderate geometric variations that simulate realistic head-position changes during MRI acquisition. Intensity-altering operations (e.g., noise injection, gamma correction) were avoided because brain-age prediction relies on subtle morphological patterns, and altering voxel intensity distributions may introduce biologically implausible aging cues.

Each original scan in the selected age ranges produced six additional training instances, improving representation of extreme ages and stabilizing regression performance across the full lifespan. To assess the impact of data augmentation on dataset balance, we compared the distribution of samples across age ranges before and after augmentation for both training and validation sets (Tables 3 and 4). In the training set, augmentation substantially increased the representation of underrepresented age groups: for example, subjects aged < 10 years increased from 138 (5.46%) to 966 (16.11%), and subjects aged $\ge$ 80 years increased from 5 (0.20%) to 35 (0.58%). Similarly, in the validation set, subjects aged $<10$ years increased from 36 (5.70%) to 252 (17.26%), and subjects aged $\ge$ 80 years increased from 1 (0.16%) to 7 (0.48%). Table 5 provides an overview of the final dataset following augmentation.

These adjustments demonstrate that data augmentation not only increased the number of training samples but also substantially reduced age imbalance, providing a more uniform distribution for model training and improving generalization, particularly for previously underrepresented age ranges.

**Table 3:** Training DataSet before and after augmentation.

Age range	Not augmented data		Augmented data	
	Data size	Size percentage	Data size	Size percentage
$age < 10$	138	5.46%	966	16.11%
$10 \le age < 20$	818	32.36%	818	13.64%
$20 \le age < 30$	1132	44.78%	1132	18.88%
$30 \le age < 40$	150	5.93%	1050	17.51%
$40 \le age < 50$	69	2.73%	483	8.06%
$50 \le age < 60$	71	2.81%	497	8.29%
$60 \le age < 70$	97	3.84%	679	11.32%
$70 \le age < 80$	48	1.90%	336	5.60%
$age \ge 80$	5	0.20%	35	0.58%
**Total**	2528	100%	5996	100%

**Table 4:** Validation DataSet before and after augmentation.

Age range	Not augmented data		Augmented data	
	Data size	Size percentage	Data size	Size percentage
$age < 10$	36	5.70%	252	17.26%
$10 \le age < 20$	211	33.39%	211	14.45%
$20 \le age < 30$	283	44.78%	283	19.38%
$30 \le age < 40$	23	3.64%	161	11.03%
$40 \le age < 50$	24	3.80%	168	11.51%
$50 \le age < 60$	18	2.85%	126	8.63%
$60 \le age < 70$	22	3.48%	154	10.55%
$70 \le age < 80$	14	2.22%	98	6.71%
$age \ge 80$	1	0.16%	7	0.48%
**Total**	632	100%	1460	100%

**Table 5:** Dataset after augmentation.

	Train data	Validation data	Test data
Size	5996	1460	791
Age Mean	33.97	33.89	24.75
Age Std	20.76	21.26	14.10
Males	3250 (54.20%)	806 (55.21%)	412 (52.09%)
Females	2746 (45.80%)	654 (44.79%)	379 (47.91%)

### MRI slices selection

**Fig. 4:**
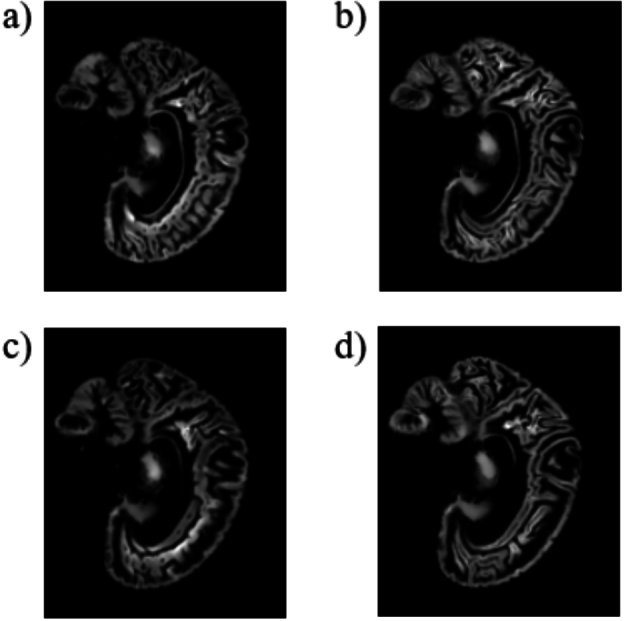
Dataset samples – (**a**, **b**) Slice 56, (**c**, **d**) Slice 66, (a, c) Age 21, (b, d) Age 31.

In the dataset, each MRI scan consists of 121 slices, each with a resolution of 145 $\times$ 121 pixels. Figure 4 presents sample slices from the dataset, where images (a) and (b) correspond to slice 56 from individuals aged 21 and 31, respectively, while images (c) and (d) represent slice 66 from the same age groups.

The superior and inferior regions of the volume contain limited cortical information, as they mainly capture skull boundaries and non-brain tissue. Following prior neuroimaging preprocessing practices reported in the literature^36^, peripheral slices with negligible gray-matter content were removed. Specifically, the first and last 18 slices were excluded from every scan, consistent with established brain-cortical preprocessing procedures^36^. This fixed criterion was applied uniformly across all subjects to ensure reproducibility and avoid subject-dependent variability.

After exclusion, 85 central slices per scan (each with a resolution of 145 $\times$ 121 pixels) were retained as model input. This step reduces noise from non-informative regions while preserving cortical and subcortical structures that carry age-related morphological patterns.

### Model tuning and optimization

To identify the optimal configuration of the proposed deep learning model, a series of experiments was performed to evaluate the influence of key architectural and regularization hyperparameters on training stability and generalization. Experiments covered convolutional, recurrent, and dense layer parameters, including the number of filters, kernel and pooling sizes, network depth, and regularization strategies. These experiments aimed to balance model capacity, overfitting control, and computational efficiency. Table 6 summarizes the main hyperparameters investigated, their tested ranges, and the configurations adopted in the final model.

**Table 6:** Experimental ranges of hyperparameters evaluated during model training.

Parameter	Range Tested	Selected Value	Observation
Filter in each CNN layer	8 to 256	64, 128, & 128	Higher filters improved representation but increased overfitting
Kernel size for each CNN layer	(1,1) to (7,7)	(5,5), (3,3), & (1,1)	Larger kernels slowed training, minor gains
Pool size in each CNN layer	(1,1) to (5,5)	(1,2)	Balanced spatial reduction and feature retention
CNN layers	2 to 5	3	Deeper networks overfit small data folds
Regularizers on Dense layer	L1, L2, & L1L2	L2	Best validation consistency
Batch size	8 to 64	32	Stable training

Table 7 presents an ablation study analyzing the impact of different architectural configurations on brain age prediction performance. The experiments varied the convolutional filter sizes, number of filters, and inclusion of LSTM or bidirectional LSTM layers. Results show that incorporating a bidirectional LSTM significantly improves predictive accuracy compared to single-LSTM configurations, underscoring the importance of capturing temporal dependencies across MRI slices. Among all tested architectures, the final model achieved the lowest mean absolute error (MAE = 3.15) and the highest coefficient of determination ($R^{2}$ = 0.86), demonstrating an effective balance between model complexity and performance.

**Table 7:** Ablation study of model architectures.

Model	CNNs Filters/Kernel Size	LSTM Units	Bidirectional LSTM Units	Description	MAE	$R^{2}$
Model 1	CNN1: 16/(3,3) CNN2: 32/(3,3) CNN3: 16/(3,3)	32	N/A	Standard LSTM-based CNN	3.7166	0.8102
Model 2	CNN1: 32/(7,7) CNN2: 64/(5,5) CNN3: 128/(3,3)	N/A	64	Deeper CNN with Bi-LSTM	3.2441	0.8647
Model 3	CNN1: 32/(5,5) CNN2: 64/(3,3) CNN3: 128/(1,1)	N/A	64	Compact kernel configuration	3.2142	0.8615
Model 4	CNN1: 64/(7,7) CNN2: 128/(5,5) CNN3: 256/(3,3)	N/A	64	High-capacity CNN	3.2326	0.8524
Model 5	CNN1: 64/(5,5) CNN2: 128/(3,3) CNN3: 256/(1,1)	N/A	64	Reduced kernel depth	3.1960	0.8564
**Final Model**	**CNN1: 64/(5,5)** **CNN2: 128/(3,3)** **CNN3: 128/(1,1)**	**N/A**	**64**	**Optimized configuration**	**3.1573**	**0.8648**

To optimize our model’s performance on the OpenBHB^22^ dataset, we carefully tuned several hyperparameters and employed effective training strategies. The Adam optimizer^37^ was chosen for its adaptive learning rate, enabling efficient convergence. The initial learning rate was set to $10^{-4}$ and was progressively reduced by a factor of 0.9 every 10 epochs to enhance stability and prevent overshooting optimal solutions^38^. The loss function used for training was Mean Absolute Error (MAE), as it provides a direct measure of prediction accuracy by evaluating the absolute difference between predicted and actual brain ages^39^. Additionally, model performance was assessed using Mean Absolute Error (MAE) and $R^{2}$ Score. MAE quantifies average prediction error, and $R^{2}$ Score evaluates how well the model explains variance in the data. Table 8 summarizes all the hyperparameters used in the experiments. Several values were adopted from previous studies, while others were determined through empirical testing to optimize model performance. These tuning strategies and evaluation methods ensured that the model effectively learned age-related patterns while minimizing errors and improving generalization across different age groups in the dataset.

All model training and hyperparameter tuning were conducted on a high-RAM CPU instance in Google Colab (51 GB memory) without GPU acceleration. Due to the slice-based architecture and inter-slice dependency modeling of high-resolution MRI data, each training epoch required approximately 50 minutes. Although this training time reflects the computational demands of the proposed framework, it demonstrates the approach’s feasibility on widely accessible CPU-only resources. GPU acceleration was not utilized due to hardware availability constraints during the experimental phase. Nevertheless, training on modern GPU hardware is expected to substantially reduce computational time and would facilitate the exploration of larger batch sizes and more complex architectural variants in future work.

**Table 8:** Hyperparameters for the proposed model.

Hyperparameter	Value	Rationale
Loss Function	Mean Absolute Error (MAE)	Robustness and interpretability in regression tasks^39^
Optimizer	Adam	Effectiveness and widespread^37^
Learning Rate	$10^{-4} \times 0.9^{{\left\lfloor n/10 \right\rfloor }^{1}}$	prevalent in prior studies^38^
Batch size	32	Effective balance between training stability, computational efficiency, and generalization performance
Number of epochs	77	Experimental results obtained
Regularization	L2 regularization on both kernel and activity	More stable and robust model, particularly important for biomedical tasks like brain age prediction^40^

^1^n: Epoch Number.

## Results

**Fig. 5:**
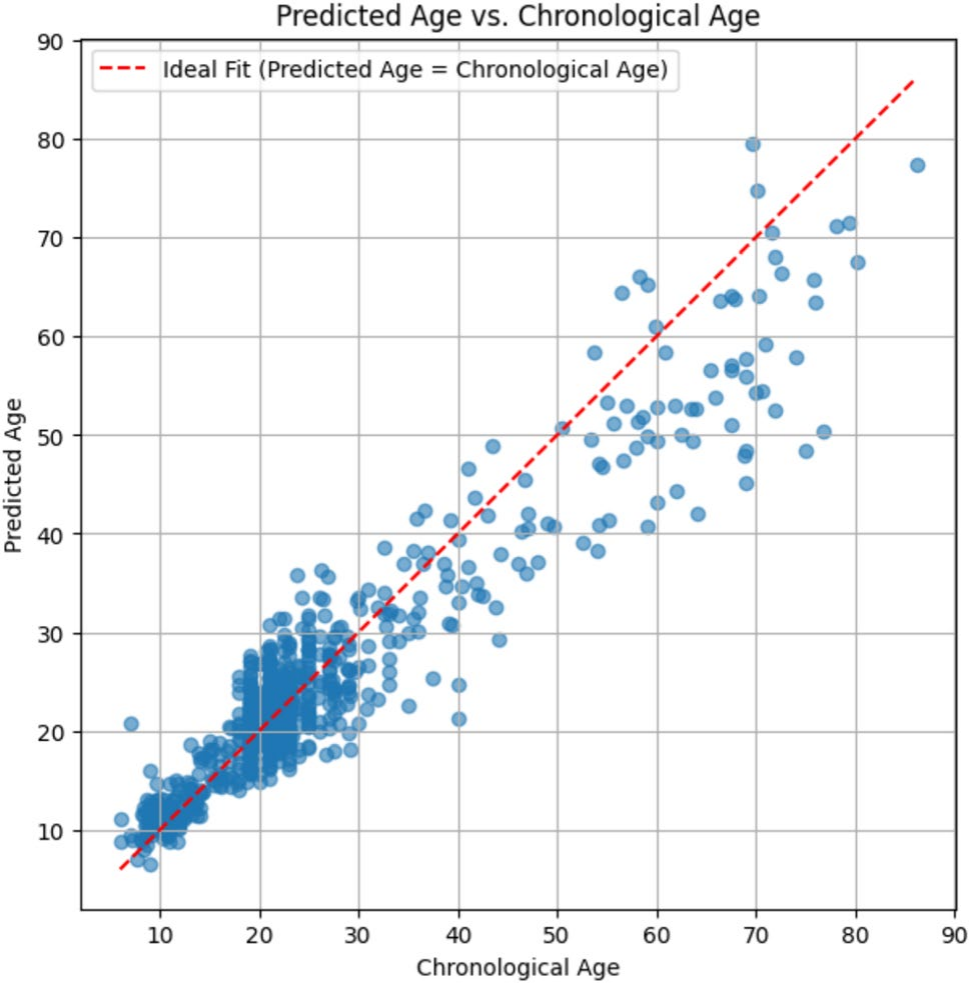
Predicted age vs. chronological age scatter plot.

Figure 5 illustrates the relationship between the predicted and chronological ages across all test subjects. Each point corresponds to an individual MRI sample, while the red dashed line represents the ideal identity line (Predicted Age = Chronological Age). The close clustering of data points around the diagonal demonstrates a strong agreement between predicted and actual ages, indicating the model’s high predictive accuracy and low mean absolute error (MAE). Slight deviations from the line, particularly at older ages, suggest minor underestimation or overestimation trends in those ranges. Overall, this pattern confirms that the proposed model effectively captures age-related structural brain features and generalizes well across the population.

**Fig. 6:**
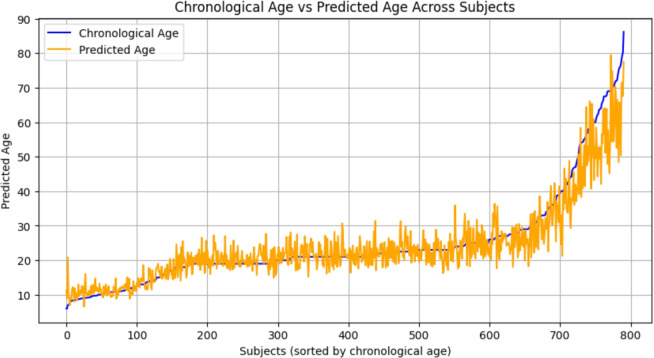
Comparison of chronological and predicted ages across subjects.

Figure 6 presents a comparative visualization of chronological and predicted ages across individual subjects. The x-axis shows subjects sorted by chronological age, and the y-axis shows age values. The blue line depicts the true chronological ages, which form a steadily increasing trend, whereas the orange line represents the model’s predicted ages, which exhibit greater fluctuation–especially among older subjects. Although the predicted values generally follow the same upward trajectory as the true ages, noticeable deviations appear at certain points. These discrepancies indicate that, while the model successfully learns the overall aging trajectory, prediction errors are more pronounced for specific individuals or age intervals.

**Fig. 7:**
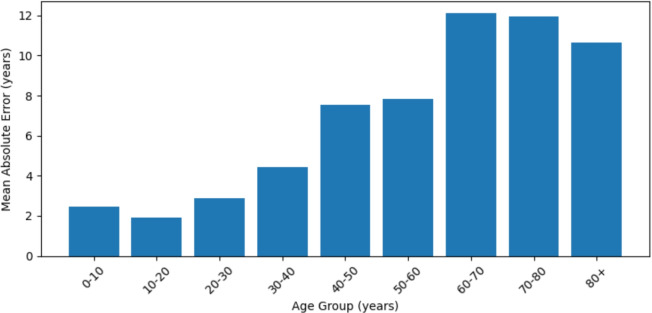
Age-stratified Mean Absolute Error (MAE) by decade.

Figure 7 illustrates the age-stratified Mean Absolute Error (MAE) of the proposed brain age prediction model across decade-based age groups. The model achieves its lowest prediction errors in younger cohorts (0–10 and 10–20 years), indicating effective learning of early brain developmental patterns. A gradual increase in MAE is observed from early adulthood (20–40 years), followed by a marked degradation in performance in older age groups. In particular, prediction errors rise sharply beyond 40 years of age and reach their highest values in the 60–70, 70–80, and 80+ cohorts. This behavior is strongly influenced by the dataset’s age distribution, as shown in Tables 3 and 4, which reveal a substantial reduction in the number of training and validation samples at higher ages, even after data augmentation. The limited representation of older subjects limits the model’s ability to fully capture heterogeneous aging-related structural changes, leading to increased prediction uncertainty. This age-dependent error pattern underscores the importance of balanced age coverage to achieve robust lifespan-wide performance.

To support the final architecture selection, an ablation study was previously conducted (see Table 7) to evaluate the impact of different CNN and LSTM configurations on prediction performance. The chosen model demonstrated the optimal balance between accuracy and computational efficiency, achieving the lowest MAE and highest $R^{2}$, and was therefore adopted for the subsequent evaluations presented in this section. Table 9 presents a comparison of various models evaluated on the OpenBHB^22^ dataset for brain age prediction, including details on dataset size, mean and standard deviation of age, and gender distribution for each study. While multiple studies have reported results across different test scenarios, our primary comparison is with the model in^31^, as both approaches use the same training and validation splits. The model in^31^ achieved a Mean Absolute Error (MAE) of 3.25 and a $R^{2}$ score of 0.90 on a custom test set comprising 20% of the dataset. In comparison, our proposed model demonstrates superior performance, achieving a lower MAE of 3.15 while maintaining a competitive $R^{2}$ of 0.86, indicating a focus on minimizing absolute prediction errors rather than maximizing explained variance. Although the $R^{2}$ value is slightly lower, this difference is relatively minor and may be attributed to $R^{2}$’s sensitivity to variance and to systematic prediction errors, particularly at age extremes. In contrast, MAE provides a more direct measure of prediction error and is widely used as a primary evaluation metric in brain age estimation studies. Therefore, the reduction in MAE suggests that the proposed model offers improved predictive accuracy.

Overall, the improved performance can be attributed to the architectural design, preprocessing strategy, and hyperparameter optimization employed in the proposed framework, which collectively enhance its ability to capture age-related neuroanatomical patterns in MRI data.

**Table 9:** Evaluation of proposed model.

Model	Dataset	Result on	Result Data Size	Result Data Age (Mean/Std)	Result Data Gender (%M/%F)	MAE	$R^{2}$
Model in^29^	OpenBHB	Training	3000	–$^{1}$	–$^{1}$	4.554	0.806
Model in^30^	OpenBHB	Internal Test	664	25.3/14.2	52.0/48.0	4.55	0.84
Model in^30^	OpenBHB	External Test	720	22.3/11.1	53.3/46.7	5.47	0.74
Model in^31^	$\hbox {OpenBHB}^{2}$	Custom $\hbox {Test}^{3}$	793	23.8/12.8	54.7/45.3	3.25	0.90
**Our Proposed Model**	$\hbox {OpenBHB}^{2}$	Custom $\hbox {Test}^{3}$	791	24.75/14.10	52.1/47.9	**3.15**	0.86

$^{1}$Demographics not available for that model.

$^{2}$Trianing dataset + Validation dataset.

$^{3}$20% of the dataset.

## Conclusion

In this study, we developed and evaluated a deep learning model for brain age prediction using the OpenBHB^22^ dataset processed with Voxel-Based Morphometry (VBM). Our approach involved several key preprocessing steps, including outlier detection and removal, dataset augmentation, and MRI slice selection, to ensure the model was trained on high-quality, well-balanced data. The proposed model was fine-tuned with an adaptive learning rate, optimized with the Adam optimizer, and evaluated using Mean Absolute Error (MAE) and the $R^{2}$ Score. The results demonstrated that our model outperformed a previously published model on the same dataset, achieving a lower MAE and improved predictive accuracy. The findings highlight the effectiveness of the preprocessing techniques in improving model robustness and generalization. By refining MRI input selection and carefully structuring the dataset, we enhanced the model’s ability to learn meaningful age-related patterns. These results contribute to the growing body of research on automated brain age prediction, which has significant implications for studying neurodegenerative diseases, cognitive aging, and early-stage abnormality detection in neurological disorders.

However, certain limitations remain. Although the OpenBHB^22^ dataset is comprehensive, broader population diversity would further enhance generalizability across different cohorts. The proposed framework relies solely on structural MRI (VBM), and the absence of multimodal imaging inputs may limit its ability to capture complementary functional and microstructural brain information. From a computational perspective, training in a CPU-based environment limited exploration of larger, more complex architectures and increased overall training time. In addition, site-related variability in scanner hardware and acquisition protocols may introduce biases that affect performance across unseen clinical centers. Although the model achieved high accuracy when trained and evaluated on the OpenBHB^22^ dataset – which includes data from 71 acquisition sites – explicit harmonization techniques were not applied. Finally, translation into clinical practice requires further validation under real-world constraints, including heterogeneous imaging protocols, computational efficiency requirements, and data privacy considerations.

Building on these findings, several concrete directions are planned. First, the proposed framework will be extended to multimodal neuroimaging by incorporating functional MRI (fMRI) and diffusion tensor imaging (DTI) to capture complementary structural and functional information. Second, harmonization strategies such as ComBat and domain-adversarial learning will be investigated to explicitly mitigate inter-site variability and further improve cross-cohort generalizability. Third, explainable AI (XAI) techniques, including saliency mapping and attention-based visualization, will be integrated to enhance interpretability and support clinical insight. Finally, advanced hyperparameter optimization, improved regularization strategies, and GPU-accelerated training will be explored to improve performance stability, scalability, and computational efficiency in larger-scale deployments.

Overall, this study presents a robust deep learning model for brain age prediction, demonstrating its potential for future clinical and research applications. The insights from this work lay the foundation for further advances in brain aging research, ultimately contributing to improved understanding and early detection of age-related neurological conditions.

## Data Availability

This study utilized the OpenBHB dataset, which is publicly available at https://dx.doi.org/10.21227/7jsg-jx57. Additional data or materials generated during this study are available from the corresponding author upon request.
